# c-KIT signaling is targeted by pathogenic *Yersinia* to suppress the host immune response

**DOI:** 10.1186/1471-2180-13-249

**Published:** 2013-11-09

**Authors:** Sofiya N Micheva-Viteva, Yulin Shou, Kristy L Nowak-Lovato, Kirk D Rector, Elizabeth Hong-Geller

**Affiliations:** 1 Bioscience Division, Los Alamos National Laboratory, Los Alamos, NM 87544, USA; 2 Chemistry Division, Los Alamos National Laboratory, Los Alamos, NM 87544, USA

**Keywords:** RNA interference, *Yersinia* infection, Host response, Signal transcription, Virulence, Host-pathogen interactions

## Abstract

**Background:**

The pathogenic *Yersinia* species exhibit a primarily extracellular lifestyle through manipulation of host signaling pathways that regulate pro-inflammatory gene expression and cytokine release. To identify host genes that are targeted by *Yersinia* during the infection process, we performed an RNA interference (RNAi) screen based on recovery of host NF-κB-mediated gene activation in response to TNF-α stimulation upon *Y. enterocolitica* infection.

**Results:**

We screened shRNAs against 782 genes in the human kinome and 26 heat shock genes, and identified 19 genes that exhibited ≥40% relative increase in NF-κB reporter gene activity. The identified genes function in multiple cellular processes including MAP and ERK signaling pathways, ion channel activity, and regulation of cell growth. Pre-treatment with small molecule inhibitors specific for the screen hits c-KIT and CKII recovered NF-κB gene activation and/or pro-inflammatory TNF-α cytokine release in multiple cell types, in response to either *Y. enterocolitica* or *Y. pestis* infection.

**Conclusions:**

We demonstrate that pathogenic *Yersinia* exploits c-KIT signaling in a T3SS-dependent manner to downregulate expression of transcription factors EGR1 and RelA/p65, and pro-inflammatory cytokines. This study is the first major functional genomics RNAi screen to elucidate virulence mechanisms of a pathogen that is primarily dependent on extracellular-directed immunomodulation of host signaling pathways for suppression of host immunity.

## Background

The genus *Yersinia* includes three human pathogens, *Y. pestis*, the etiological agent of plague via intradermal fleabites or inhalation, and *Y. pseudotuberculosis* and *Y. enterocolitica*, which cause self-limiting enteric disease by the oral route. In spite of the differences in route of infection and severity of disease, the three species share similar pathogenic mechanisms, primarily the ~70 kb virulence plasmid (pCD1 in *Y. pestis* and pYV in *Y. pseudotuberculosis* and *Y. enterocolitica*) that encodes for the Type III secretion system (T3SS) [1]. Upon contact with host cells and a shift to host temperature of 37°C, *Yersinia* induces T3SS expression to translocate *Yersinia* outer proteins (Yops) into the host cytosol to modulate the host immune response and promote pathogen survival [2].

All three *Yersinia* species target the lymphoid system during infection and replicate in lymphatic tissue as aggregates of extracellular bacteria [3,4]. *Yersinia* strains that lack pCD1/pYV do not replicate extracellularly and have been shown to be contained within granulomas that are eventually eliminated [4]. *Yersinia* are unusual amongst other Gram-negative bacteria that express the T3SS, in that they do not actively induce phagocytosis for entry and intracellular growth in the host [5]. Instead, *Yersinia* inject several Yops, including YopH, E, and T, to disrupt the host actin cytoskeleton and resist uptake via phagocytosis by neutrophils. Although pathogenic *Yersinia* have been reported to multiply within macrophages early in the infection process [6,7], *Y. pestis* exponential growth occurs primarily in the extracellular phase, causing acute septicemia with blood counts as high as 10^8^ CFU/ml [8]. Thus, in order to establish successful infection, *Yersinia* is dependent on targeting multiple host signaling pathways to evade host immune defense and induce host cell death. For example, YopP/J functions as a deubiquitinating protease and acetyltransferase to inhibit both the host NF-κB and mitogen-activated protein kinase (MAPK) signaling pathways, leading to a block in cytokine secretion and apoptosis of host macrophages [9-11]. Although discovery of Yop effector targets have begun to clarify mechanisms of *Yersinia* virulence, it is likely the case that additional host targets remain to be defined. Identification of host cell factors that are targeted by *Yersinia* during infection would provide valuable molecular insights in understanding *Yersinia* pathogenesis, and ultimately, in designing effective host-targeted therapies and antimicrobial agents.

In order to systematically identify novel host targets required for *Yersinia* infection, we performed an RNAi screen using a short hairpin RNA (shRNA) kinome library. The development of RNAi approaches has greatly enabled the examination of the roles of individual human genes by specific gene silencing [12]. Both small and large-scale RNAi screens have been applied to the discovery of host targets in response to infection by intracellular pathogens, including *S. typhimurium*[13], *M. tuberculosis*[14], and *L. monocytogenes*[15], and the HIV [16-18], HCV [19,20], and influenza [21,22] viruses. Our shRNA screen is based on the recovery of NF-κB activation following *Y. enterocolitica* infection of HEK-293 cells. NF-κB controls expression of genes involved in the inflammatory response, including TNF-α, IL-1, IL-6, IL-12, and MIP1β, and thus plays a critical role in the clearance of the bacteria by the immune response. We identified 19 host genes that are targeted by *Y. enterocolitica* to inhibit NF-κB-regulated gene expression and validated their role in host cells infected with *Y. pestis*, in addition to *Y. enterocolitica.* We also describe a novel c-KIT-EGR1 host signaling pathway that is targeted by *Yersinia* during the infection process. To the best of our knowledge, this is the first major RNAi effort to screen for host targets in response to a predominantly extracellular pathogen.

## Results

### RNAi screen to identify host cell factors that are required for *Yersinia*-mediated inhibition of NF-κB-driven gene expression

**Figure 1:**
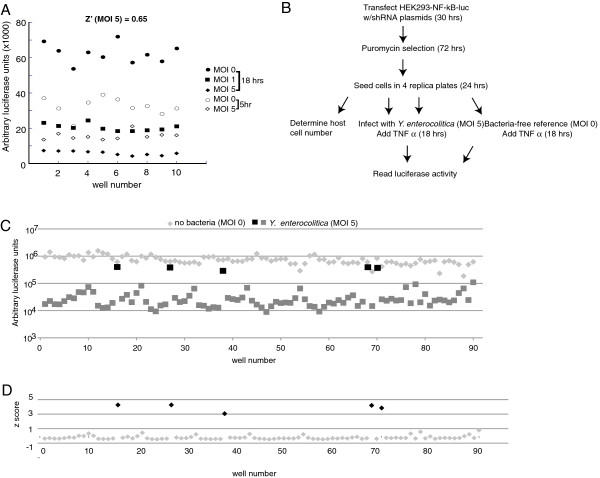
**Assay optimization and shRNA screen design. (A)***Y. enterocolitica* WA inhibits NF-κB signaling through TNF-R. RE-luc2P-HEK293 cells were infected with *Y. enterocolitica* WA, at either MOI 0 (circles), 1 (square), or 5 (diamonds), in a 96-well plate. Cells were stimulated either 5 h post infection with 10 ng/ml TNF-α and incubated for an additional 18 h (closed symbols) or 2 h post-infection with 10 ng/ml TNF-α and incubated for another 5 hrs (open symbols). The arbitrary luciferase activity per well from a representative of two experiments (n=10/expt) is presented. Z’ was calculated using the SD and mean of luciferase activity from cells infected with *Y. enterocolitica* WA at MOI 5 versus cells not treated with bacteria (MOI 0) at each time point [24]. The best Z’ value 0.65 was obtained for the 18 h time point at MOI 5. **(B)** For the shRNA screen, the kinome plasmid library was transfected in 96 well format, and cells were subjected to puromycin selection to enrich for populations expressing the inhibitory sequences. Chloramphenicol (170 μg/ml) was added 1 h post-infection to control extracellular bacteria counts. At 5 h post-infection, 10 ng/ml TNF-α was added to the cells and NF-κB-driven luciferase activity was determined 18 h later. **(C)** The hit selection cut-off was determined as ≥40% direct recovery in luciferase signal of *Yersinia*-infected cells (black squares) relative to non-hits (gray squares) and bacteria free samples (light gray diamonds). **(D)** The statistical significance of assay hit selection was evaluated using a standard z-score. Genes in which silencing resulted in assay reads with a score ≥3 standard deviations above the assay mean score were considered to be true hits with a strong effect on *Yersinia*-driven inhibition of NF-κB signaling (shown in black diamonds), compared to non-hits (gray diamonds).

We conducted a functional genomic screen using 2503 shRNA hairpins targeting 782 human kinase and kinase-related genes to identify host factors that inhibit NF-κB-mediated gene expression by pathogenic *Yersinia*. The screen was performed using the highly-virulent *Y. enterocolitica* WA strain, which has been shown to impair NF-κB activation and pro-inflammatory cytokine production more efficiently than virulent *Y. pestis* strains and induces a strong apoptotic effect on host cells [23]. To maximize assay sensitivity and noise reduction for the screen, we stimulated the HEK293 cell line with the inflammatory mediator TNF-α, resulting in ~70-fold induction of NF-κB reporter gene activity, an excellent signal-to-noise ratio for a high throughput screen (HTS) (Figure 1A). We calculated the Z-factor (Z’) to be ~0.65 upon infection of HEK293 at MOI 5 for 5 hrs, followed by 18 h of TNF-α stimulation. Z’ is a statistical evaluation of HTS performance and reflects the robustness and reliability of the assay. Z’ ≥ 0.5 is equivalent to ≥ 12 standard deviations between the positive and negative controls and represents excellent assay parameters (see Methods for a more detailed description of Z’) [24]. We designed our screen (Figure 1B) to select for shRNAs that increased NF-κB-driven luciferase activity ≥40% compared to the mean of all assay reads in *Y. enterocolitica*-infected, TNF-α stimulated cells for each plate. (Figure 1C, black squares compared to grey squares) Additionally, we applied a standard z-score method to identify shRNAs that produced a statistically-significant recovery (z score ≥3) of luciferase activity (Figure 1D, black diamonds).

We identified 18 kinase genes, that when silenced, led to recovery of NF-κB-mediated luciferase activity in response to *Y. enterocolitica* infection (Table 1). The screen identified genes that function in different cellular processes, including signal transduction (e.g., MAP kinases, CKII), cytoskeleton dynamics (e.g. c-KIT, ABL, PAK4), and regulation of ion channel activity (e.g. SGK, WNK). In addition to the kinase shRNA library, we screened a collection of 62 shRNA constructs that targeted 26 genes annotated for chaperone activity to determine whether the heat shock, protein folding, and stress response machinery is required for successful *Yersinia* infection. We found that silencing of HSPH1, caused recovery of NF-κB regulated gene expression in response to *Y. enterocolitica* infection (Table 1).

**Table 1:** **Host genes identified from shRNAmir kinome screen required for *Y. enterocolitica *** **mediated knock-down of NF-κB activation**

**Gene name**	**Accession #**	**Function**
**ABL**	NM_005157	Regulation of cell differentiation and adhesion
ACTR-IIB	NM_001106	Activin and myostatin signaling involved in apoptosis, inflammation
**CKII**	NM_177560	Regulation of metabolic pathways, signal transduction, transcription
ERK4/MAPK7	NM_139033	Signaling of receptor kinases and G protein-coupled receptors
**GNE/DMRV**	NM_005476	Rate-limiting enzyme in sialic acid biosynthesis
**HSPH1**	NM_006644	Reduces protein aggregation and cytotoxicity through induction of HSP70
**c-KIT**	NM_000222	Differentiation of hematopoietic progenitor cells and mast stem cells
**MAP3K3**	NM_002401	Regulation of NF-κB gene expression
MEK1	NM_002755	MAPK signal transduction pathways involved in transcription regulation
MONaKA/PXK	NM_017771	Binds to and modulates Na,K-ATPase enzymatic and ion pump activities
**NIK/MAP3K14**	NM_003954	Activates NF-κB in response to TNFR and IL-1R signaling
**PAK4**	NM_005884	Inactivates actin binding/depolymerizing factor cofilin
PIK3R2	NM_005027	PI3K/Akt signaling associated with cell survival, proliferation
**PI3-K-C2A**	NM_002645	Member of the PI3K family, potential role in integrin-dependent signaling
**SGK1**	NM_005627	Regulation of cell stress response through activation of ion channels
SGK2	NM_170693	Regulates ion channels in response to signals that activate PI3K
ULK2	NM_014683	Stress response, mediates mTOR signaling in autophagy
**WNK1**	NM_018979	Regulator of blood pressure by activating ion channel transporters
WNK3	NM_020922	Positive regulator of the transcellular Ca2+ transport pathway

Genes in **bold** were selected for further validation.

### Validation of candidate ‘hits’ from RNAi screen

**Figure 2:**
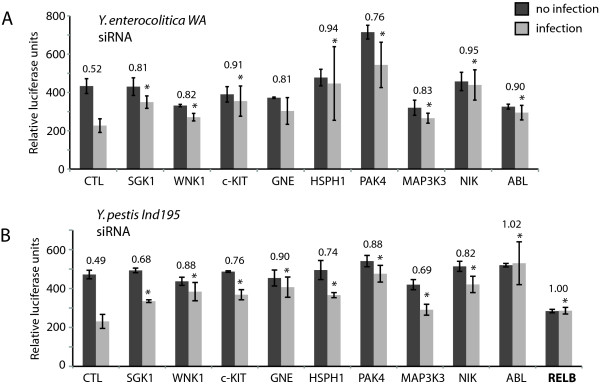
**Secondary RNAi assays for validation of host factors required for *Yersinia*-mediated suppression of NF-κB signaling.** RE-luc2P-HEK293 cells (20,000/well) were transfected with a 10 nM siRNA pool of four sequences per target gene in a 96-well plate and cultured for 72 h prior to **(A)***Y. enterocolitica* WA or **(B)***Y. pestis* Ind195 infection at MOI 2 and 10, respectively. Infection was stopped 1 h post-bacteria exposure by addition of 170 μg/ml chloramphenicol in the culture media. Cells were stimulated 5 h post-infection with 10 ng/ml TNF-α and were further cultured for 18 h. Cells were lysed and luciferase activity was measured to determine the percentage recovery of NF-κB regulated gene expression relative to that of uninfected cells. NF-κB-regulated luciferase activity was normalized to the RE-luc2P-HEK293 cell titer for each sample to obtain relative luciferase units. Numbers above the column data represent the ratio of luciferase activity in bacteria-infected versus uninfected cells. A “*” denotes significant (p≤0.05) recovery of reporter activity for targeting siRNAs compared to the control non-targeting siRNA (CTL)-treated cells infected with bacteria. Data was obtained from four independent experiments performed in duplicate.

We selected 9 genes, SGK1, WNK1, c-KIT, GNE1, HSPH1, PAK4, MAP3K3, NIK/MAP3K14, and ABL, representative of different cellular pathways, for further validation studies. We performed a secondary RNAi screen using a pool of siRNA duplexes that targeted four different sequences per gene. Introduction of the siRNA duplexes into RE-luc2P-HEK293 cells resulted in ≥ 70% reduction in cognate gene mRNA levels (data not shown) and reiterated the ≥40% recovery of TNF-α-induced NF-κB gene expression in response to *Y. enterocolitica*, as previously seen in the original shRNA screen (Figure 2A). Silencing of all nine genes increased the ratio of NF-κB-driven luciferase activity between infected and uninfected cells (0.81 to 0.94), when compared to HEK293 cells expressing a control (CTL) siRNA (0.52). Similarly, siRNA silencing increased the ratio (0.68 to 1.02) of NF-κB expression between *Y. pestis* Ind195-infected and uninfected cells compared to the control sample (0.49), suggesting that many of the host genes identified from the screen are also targeted by *Y. pestis* during onset of plague (Figure 2B).

To determine whether siRNA treatment itself significantly dampened NF-κB-regulated gene expression, we examined luciferase activity in cells treated with siRNAs against RelB, a member of the NF-κB family. In the absence of infection, luciferase activity was decreased ~2-fold in cells treated with siRNAs against RelB, compared to the other siRNA-treated cells. (Figure 2B, dark grey bars, RelB in bold) Infection with the virulent *Y. pestis* Ind195 strain produced no further change in luciferase expression (Figure 2B, light grey bars, RelB), indicating that a basal level of luciferase activity had been reached in cells depleted of RelB. Our data suggest that siRNA treatment alone did not significantly manipulate NF-κB activity.

### Use of small molecule inhibitors to validate kinase function in *Yersinia*-mediated inhibition of NF-κB activation and cytokine production

**Figure 3:**
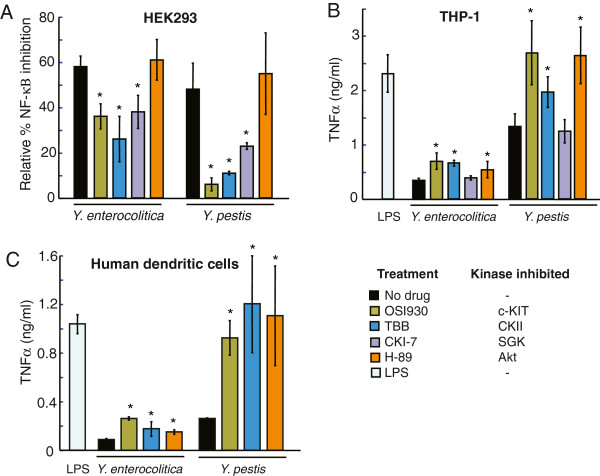
**Analysis of host kinase function in *Yersinia*-mediated immune suppression using small molecule inhibitors. (A)** RE-luc2P-HEK293 cells were pretreated with 1μM OSI-930 (green), 20μM TBB (blue), 10μM CKI-7 (purple), or 10μM H-89 (orange) for 16 h and infected with *Y. enterocolitica* WA or *Y. pestis* Ind195 at MOI 1 and 20, respectively, for 1 h. Following stimulation with 10 ng/ml TNF-α at 5 h post-infection, luciferase activity was measured 24 h post-infection. Results were determined from two independent experiments performed in triplicate. A ‘*” denotes that the % NF-κβ inhibition using the inhibitors was significantly different (p<0.05) compared to the no drug control (black). The relative NF-κB inhibition by *Yersinia* infection was determined as a percentage of luciferase activity in bacteria-infected cells relative to luciferase activity in bacteria-free control cells. **(B)** THP-1 cells were pretreated with the small molecules and infected with *Y. enterocolitica* WA or *Y. pestis* Ind195 at MOI 5 and 20, respectively, for 1 h. TNF-α levels were determined by ELISA on conditioned media collected 24 h post-infection. Results were determined from two representative independent experiments performed in quadruplicate. A ‘*” denotes that TNF-α release using inhibitors was significantly different (p<0.05) compared to the no drug control. Cytokine release in response to purified LPS from *E. coli* 055:B5 (5μg/ml, light blue) was used as a control for pro-inflammatory mediator signaling. **(C)** Normal HDC were pre-treated with the small molecules for 18 h prior to infection with *Y. enterocolitica* WA or *Y. pestis* KIM5-. Bacterial infection was stopped 1 h post-infection with 170 μg/ml chloramphenicol. TNF-α levels were determined by ELISA on conditioned media collected 24 h post-infection. Statistical analysis was performed on data from 3 experiments performed in quadruplicate. TNF-α release in response to all inhibitor treatments were statistically significant (p<0.05) compared to no drug controls.

We selected three kinases, c-KIT, CKII, and SGK1, to further validate their functions in *Yersinia*-mediated NF-κB inhibition using small molecule inhibitors (Figure 3). None of the tested kinase inhibitors induced activation of NF-κB-regulated gene expression in uninfected controls or affected *Yersinia* growth in host media (data not shown). The cell surface receptor tyrosine kinase c-KIT, also known as stem cell growth factor receptor CD117, is expressed predominantly in progenitor hematopoietic cells and mast cells. Upon stem cell factor (SCF) ligand binding, c-KIT triggers multiple signaling cascades, including PI3K/AKT, Ras/ERK, and JNK, which are essential for regulating proliferation, survival and cell differentiation [25]. Incubation of *Y. enterocolitica*- or *Y. pestis*-infected RE-luc2P-HEK293 cells with OSI-930, a highly-specific c-KIT inhibitor, led to rescue of TNF-α-induced NF-κB activation, compared to no drug controls. (Figure 3A, green vs black bars) Treatment of the monocytic cell line THP-1 or primary normal human dendritic cells (NHDC) with OSI-930 induced a similar protective effect against *Yersinia*-mediated suppression of TNF-α secretion, as measured by ELISA, indicating that c-KIT is required for *Yersinia*-induced repression of pro-inflammatory cytokine release (Figure 3B and C, green vs black bars).

We also tested the effect of the small molecule TBB, an inhibitor of the CKII serine kinase, which functions in cell stress response, cell cycle and cell growth regulation by activation of IKK. CKII also regulates expression of HSPH1, another stress response gene identified in our shRNA screen [26]. Similar to OSI930, pretreatment of RE-luc2P-HEK293, THP-1, and NHDC cells with TBB resulted in higher levels of NF-κB-regulated gene expression and TNF-α release compared to a no drug control, in response to both *Y. enterocolitica* and *Y. pestis* infection (Figure 3A-C, blue vs black bars).

The small molecule CKI-7 was used to validate the role of SGK1 (serum and glucocorticoid-inducible kinase 1) on NF-κB-regulated gene expression in response to *Yersinia* infection. SGK1 is a serine/threonine kinase that functions in cellular stress response and regulates activity of the epithelial sodium channel ENaC [27,28], a function shared with WNK1, another kinase identified from the shRNA screen. Incubation of RE-luc2P-HEK293 cells with CKI-7 resulted in increased NF-κB-mediated luciferase activity upon exposure of *Y. enterocolitica* and *Y. pestis*-infected cells to TNF-α (Figure 3A, purple vs black bars). However, CKI-7 did not lead to increased TNF-α release in *Yersinia*-infected THP-1 cells (Figure 3B, purple vs black bars). This finding is consistent with the tissue-specific expression profile of SGK1 in epithelial cells such as HEK293, but not in monocyte-like THP-1 cells [29].

Finally, we also tested the effect of H-89, a small molecule inhibitor of AKT, a downstream mediator of the PI3K pathway that plays an essential role in cell survival, migration and adhesion. Although AKT itself was not classified as a hit in the shRNA screen, we did identify PIK3R2, a regulatory subunit of PI3K, which acts directly upstream of AKT. Furthermore, AKT was previously identified as essential for intracellular growth of another T3SS pathogen, *S. typhimurium*[13]. Pre-treatment of RE-luc2P-HEK293 cells with H-89 had no effect on NF-κB-regulated luciferase activity in response to either *Y. enterocolitica* or *Y. pestis* infection (Figure 3A, orange vs black bars). However, H-89 induced a significant increase of TNF-α production in THP1 cells and NHDC infected with either *Y. enterocolitica* or *Y. pestis*, compared to untreated cells. (Figure 3B-C, orange vs black bars) These cell-type specific effects of SGK1 and PI3K/AKT likely reflect the different host cell tropism, from epithelial to macrophage cells, exhibited by *Yersinia*.

### Pathogenic *Yersinia* exploit host pathways regulated by the receptor tyrosine kinase c-KIT to suppress inflammatory cytokine release

**Figure 4:**
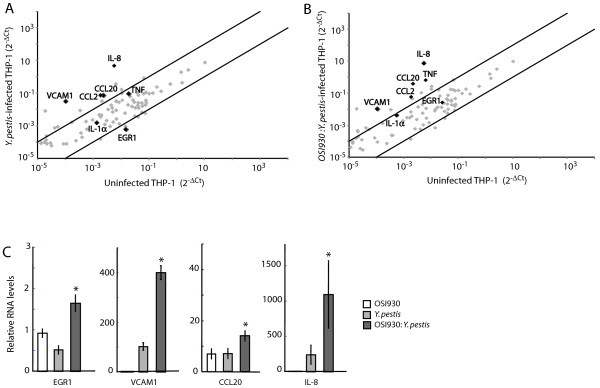
**Pathogenic *Yersinia *requires c-KIT activity for suppression of transcription factor and pro-inflammatory cytokine expression. (A-B)** Analysis of signal transduction pathways in *Y. pestis*-infected THP1 cells in absence of c-KIT. THP1 cells untreated or pre-treated with 1μM OSI-930 for 18 h were infected with *Y. pestis* Ind195 at MOI 20 for 1h. RNA was isolated, converted to cDNA, and applied to a RT Profiler PCR Array for human signal transduction pathway expression analysis. Dot plots compare gene expression profiles between uninfected THP-1 cells and **(A)***Y. pestis* Ind195-infected THP-1 cells or (**B**) OSI-930-pretreated, *Y. pestis* Ind195 infected THP-1 cells. Genes that do not exhibit significant expression changes between the control and experimental samples are concentrated between the two black lines. The black lines define the assay cut-off of 3-fold induction or 70% reduction of transcript levels. Genes of interest are highlighted in black. **(C)** Inhibition of c-KIT recovers EGR1, chemokine, and cell adhesion transcript levels in pathogenic *Yersinia*-infected THP1 cells. THP1 cells were pre-treated with 1μM OSI-930 for 18 h or were left untreated prior to infection with *Y. pestis* Ind195 at MOI 10 for 1 h. EGR1, VCAM1, CCL20, and IL-8 mRNA levels were determined by Taqman qPCR using total RNA isolated 24 h post-infection. Depicted RNA levels are relative to untreated THP1 control samples and were calculated using the 2^-ΔΔCt^ formula. A ‘*” denotes that relative RNA levels were significantly different (p<0.05) compared to infected cells untreated with OSI930. Data is shown from three independent infection experiments performed in duplicate.

We next assessed the effect of c-KIT signaling on the expression profile of 84 human inflammatory genes in *Y. pestis*-infected THP-1 cells. We observed >3-fold upregulation of several chemokines, including IL-8, CCL20, CCL2, and cell adhesion gene VCAM1 in *Y. pestis*-infected THP-1 cells compared to uninfected cells (Figure 4A). In contrast, expression of the early growth response 1 transcription factor (EGR1) was downregulated >70% in cells infected with *Y. pestis*. EGR1 has been previously found to regulate transcription of several chemokines (e.g. IL-8, CCL2) and cytokines (e.g TNF-α, IL-6), and to confer responsiveness to IL-1 and TNF signaling [30,31]. Abrogation of c-KIT signaling by OSI-930 recovered EGR-1 levels and resulted in a further increase in IL-8, CCL20, IL-1α, and TNF expression, in THP-1 cells infected with *Y. pestis* compared to untreated cells (Figure 4B).

To further explore whether c-KIT function can regulate EGR1 and downstream inflammatory gene expression, we examined the effect of OSI-930 treatment on EGR1, VCAM1, CCL20, and IL-8 gene expression in *Y. pestis*-infected THP-1 cells using qPCR (Figure 4C). Inhibition of c-KIT kinase activity by OSI-930 (Figure 4C, dark gray bar) restored EGR1 transcription >2-fold in *Y. pestis*-infected THP-1 cells compared to infected cells with functional c-KIT (Figure 4C, light gray bar). Similarly, OSI-930 treatment induced VCAM1, CCL20, and IL-8 transcription upon bacterial infection (Figure 4C, dark vs. light gray bars), suggesting that c-KIT function is required for the inhibition of key cytokines and adhesion molecules by pathogenic *Yersinia*. Notably, treatment of THP-1 cells with OSI-930 alone did not significantly change EGR1 transcript levels (Figure 4C, white bar), indicating that pharmacological inhibition of c-KIT did not initiate a non-specific immune response mediated by EGR1 in the absence of bacterial infection. Collectively, these findings suggest that there is a link between c-KIT function and suppression of the host immune response by pathogenic *Yersinia* and that transcriptional inhibition of EGR1 by *Yersinia* is dependent on c-KIT function.

**Figure 5:**
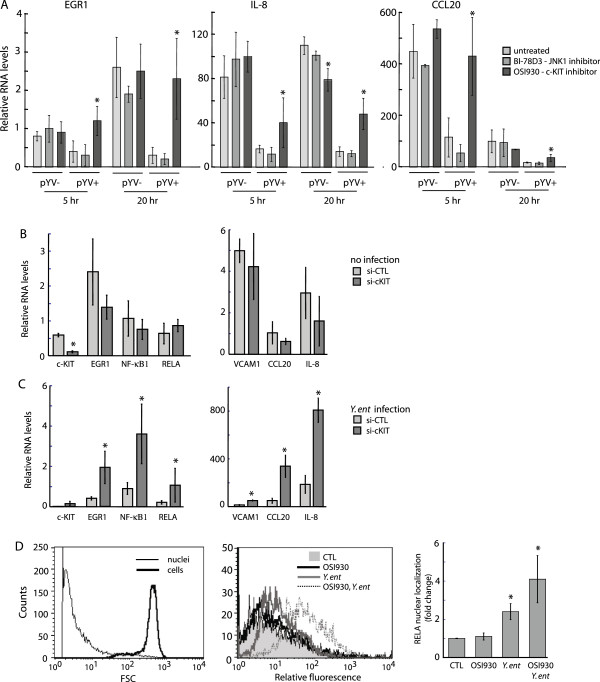
**c-KIT signaling is targeted by *Yersinia *T3SS to suppress pro-inflammatory immune response. (A)** THP1 cells were pre-treated with 1 μM OSI-930 or 1 μM BI-78D3 for 18 h or untreated prior to infection with *Y. enterocolitica* (pYV+) and *Y. enterocolitica* (pYV-) at MOI 2. The RNA levels are presented as fold change versus untreated THP1. Data is shown from three independent infection experiments performed in duplicate. A ‘*” denotes that relative RNA levels were significantly different (p<0.05) in OSI930-treated cells compared to untreated or BI-78D3-treated cells. **(B)** THP-1 cells were transfected with 50 nM siRNA targeting c-KIT or control (si-CTL) and incubated for 48 h. RNA levels are presented relative to transcript levels in siRNA-treated versus untreated THP-1. Data is shown from two representative experiments. A ‘*” denotes that relative RNA levels were significantly different (p<0.05) in si-cKIT-treated cells compared to si-CTL-treated cells. **(C)** THP-1 cells were transfected with 50 nM siRNA against c-KIT (si-cKIT) or control (si-CTL) siRNA and incubated for 72 h prior to infection with *Y. enterocolitica* WA at MOI 2 for 1 h. Gene transcript levels are depicted as a relative ratio to uninfected siRNA-treated THP-1 cells. Data is shown from three independent experiments performed in duplicate. A ‘*” denotes that relative RNA levels of immune genes were significantly different (p<0.05) in si-cKIT-treated cells compared to si-CTL-treated cells. **(D)** THP-1 cells, untreated or pretreated with 1μM OSI-930 for 5 h, were infected with *Y. enterocolitica* WA at MOI 40 for 45 min. Cell nuclei were purified, labeled with mouse anti-NF-κB RelA, and analyzed by flow cytometry. (left panel) The mean channel fluorescence was used to determine the fold change of RelA in the nuclei of *Yersinia*-infected compared to untreated THP-1 cells (middle panel). The statistical data was derived from two independent experiments (right panel).

**Figure 6:**
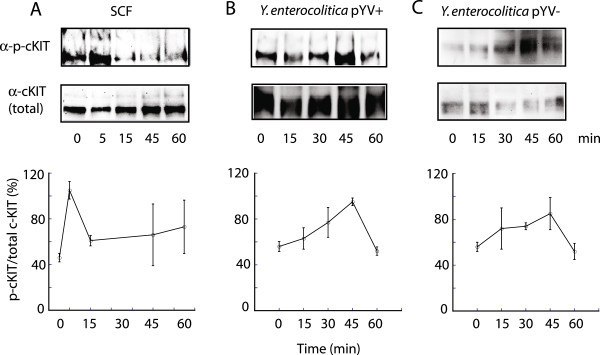
***Yersinia *infection activates c-KIT tyrosine phosphorylation.** THP-1 cells were stimulated with **(A)** SCF (50 ng/ml) or infected with **(B)***Y. enterocolitica* pYV+ or **(C)***Y. enterocolitica* pYV- at MOI 40 for the indicated time points. Cell lysates were immunoprecipitated with anti-c-KIT antibody followed by Protein A Sepharose and were resolved by 8% SDS-PAGE. Western blots were probed with anti-c-KIT and p-Tyr (PY20) antibodies. Results from three independent experiments were quantified and are presented as percentage of phosphorylated versus total c-KIT.

We next studied the role of *Yersinia* T3SS in suppression of the host immune response via c-KIT signaling. The expression profiles of EGR1, IL-8, and CCL20 were compared in THP-1 cells infected with pathogenic *Y. enterocolitica* WA and its non-pathogenic counterpart, *Y. enterocolitica* WA-01 (pYV-), cured of the pYV virulence plasmid (Figure 5A). Inhibition of c-KIT with OSI930 fully restored EGR1 levels in cells infected with virulent *Y. enterocolitica* and significantly recovered transcription of IL-8 and CCL20 at 5 h and 20 h post-infection (Figure 5A, dark grey bars). In contrast, we did not observe any significant effect by the c-KIT inhibitor OSI930 on EGR1, IL-8, and CCL20 transcription in THP-1 cells exposed to pYV- *Y. enterocolitica*. Inhibition of JNK1, acting downstream of c-KIT signaling, with the small molecule BI-78D3 [30,32] did not exhibit any protective effect on gene transcription (Figure 5A, medium grey bars) at either time point of bacterial infection, compared to drug-free cells (Figure 6A, light grey bars). Since accumulation of YopJ/P in host cells upon *Yersinia* infection has been previously linked to cell death via activation of apoptotic pathways, we assessed cell viability at various MOIs. We registered no decrease in cell viability in drug-free cells or cells treated with the JNK1 inhibitor, even after 20 h post-infection of THP-1 cells with virulent *Y.entorocolitica* at MOI 2 of the assay. (data not shown) Taken together, these findings indicate that c-KIT function is exploited by *Yersinia* T3SS to suppress production of key transcription factors and cytokines involved in the regulation of the host immune response.

We also show that ~95% depletion of c-KIT transcript levels by siRNA treatment (Figure 5B) rescued EGR1, VCAM1, CCL20, and IL-8 gene expression in response to *Y. enterocolitica* WA infection in THP-1 cells, compared to infected control cells treated with non-targeting siRNA (si-CTL) (Figure 5C). Similarly, expression levels of the NF-κB transcription factors, NF-κB1/p50 and RelA/p65, were recovered in c-KIT-silenced cells in response to *Y. enterocolitica* WA infection. In the absence of infection, silencing of c-KIT expression by siRNA did not induce any significant change in the expression levels of EGR1 or the tested cytokines and transcription factors (Figure 5B).

To further investigate the interplay between c-KIT signaling and pathogenic *Yersinia*, we measured RelA levels in purified nuclei isolated from untreated or *Y. enterocolitica*-infected THP-1 cells (Figure 5D, left panel). In response to inflammatory stimuli, RelA is normally released from its cytoplasmic inhibitor, IκBα, and transported to the nucleus to modulate gene expression [33]. Based on flow cytometric analysis, RelA protein levels were shown to increase by ~2-fold in the nuclei of THP-1 cells infected with *Y. enterocolitica* WA, compared to uninfected cells. (Figure 5D, middle and right panels) Interestingly, pre-treatment of THP-1 cells with OSI-930 led to a higher 4-fold increase of nuclear RelA levels, suggesting that *Yersinia* targets the c-KIT signaling pathway to suppress post-transcriptional activation of RelA. Collectively, our data demonstrate that virulent *Yersinia* inhibits both transcription and post-transcriptional regulation of key inflammatory proteins via the c-KIT signaling pathway.

### c-KIT phosphorylation is induced upon *Yersinia* infection independently of T3SS

We next investigated c-KIT phosphorylation to assess kinase activation in response to *Yersinia* infection. The binding of natural ligand SCF to c-KIT has been shown to induce receptor dimerization, rapid auto-phosphorylation of tyrosine residues in the intracellular domain, and subsequent recruitment of signaling proteins to activate multiple downstream pathways [34,35]. We examined c-KIT phosphorylation in THP1 cells using Western blots, in response to infection with both *Y. enterocolitica* virulent (pYV+) and attenuated (pYV-) strains (Figure 6) c-KIT exhibited maximal phosphorylation at ~45 min post-infection in both *Y. enterocolitica* strains (Figure 6B and C), compared to SCF-induced phosphorylation, which peaked at 5 min (Figure 6A), demonstrating that *Yersinia* LPS or other surface molecule can trigger c-KIT signaling, albeit at a delayed rate. This delayed phosphorylation response to pathogen exposure may stem from the time needed for bacterial chemotaxis and adhesion to host cells prior to activation of host signaling pathways.

### Differential c-KIT expression at the cell surface in human dendritic cells

**Figure 7:**
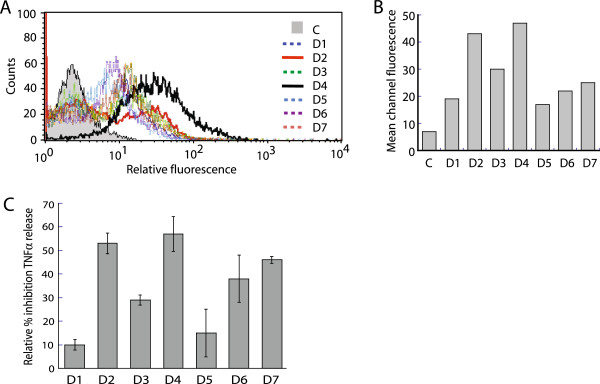
**Differential response to *Y. pestis *infection in human dendritic cells correlates with naturally-expressed c-KIT levels. (A)** Differential expression of c-KIT in human dendritic cells. NHDCs (20,000) from seven different donors (D1-7) were cultured in LGM-3 for 4 days. Both adherent and suspension cells were collected, fixed, labeled with (PE)-conjugated c-KIT (Ab81) antibody, and subjected to flow cytometry analysis. 10,000 cells were acquired to generate histograms and a bar graph **(B)** that depict fluorescence intensity distribution and mean channel fluorescence intensity. The control sample (C) was generated from a pool of unlabeled NHDC from the seven donors. **(C)** NHDCs that express high levels of c-KIT exhibit increased inhibition of TNF-α release upon *Y. pestis* infection. NHDCs from seven donors were cultured in LGM-3 for 4 days prior to treatment. Cells from a single donor were plated in 6 replicates (in a 24-well cluster dish): 2 wells were treated with LPS (*E. coli* 055:B5, 5 μg/ml) and 4 wells received *Y. pestis* Ind195 at MOI 20. The inhibition of TNF-α production by *Y. pestis*-infected cells was determined relative to LPS-treated cells for each donor. The data presented was generated from an average of four replicates of *Y. pestis*-infected cells versus the average of two replicates treated with LPS. The ELISA for each experimental sample was performed in triplicate.

To determine whether there is a link between c-KIT expression levels and host immune response, we investigated the effect of pathogenic *Yersinia* infection on pro-inflammatory cytokine production in human dendritic cells expressing naturally varying levels of c-KIT. We obtained populations of mature NHDC from seven independent human donors and compared the expression levels of c-KIT using flow cytometry with fluorescently-labeled c-KIT antibody. Two out of seven donors (D2 and D4) expressed ~2-fold higher c-KIT levels (Figure 7A and B) compared to the remaining 5 donors (D1, D3, D5-7). The NHDCs from D2 and D4 also exhibited greater relative inhibition of TNF-α release upon infection with *Y. pestis*, compared to the other donor NHDCs (Figure 7C), demonstrating that increased c-KIT expression is associated with increased suppression of pro-inflammatory cytokine release during *Yersinia* infection. These findings are consistent with the increased production of TNF-α during OSI-930 treatment of *Yersinia*-infected THP-1 and NHDC cells (Figure 3), and suggest that c-KIT may be a potential host biomarker for susceptibility to *Yersinia*–mediated suppression of innate immune response.

## Discussion

We have performed a RNAi screen to identify host genes targeted by a primarily extracellular pathogen, *Yersinia*. Most of the identified genes, including c-KIT, SGK, and CKII, have not been previously linked to pathogen infection, and thus reveal novel mechanisms of virulence and host immunity in response to *Yersinia* infection. Although the RNAi screen was based on *Y. enterocolitica* infection, the majority of validated hits were also required for NF-κB inhibition by *Y. pestis*. Given the genomic conservation between *Y. enterocolitica* and *Y. pestis*, the overlapping gene hits are likely to function in host signaling pathways impacted by common *Yersinia* pathogenesis mechanisms, such as the T3SS.

We had originally attempted to optimize a RNAi screen based on *Y. pestis* infection, but were unable to establish a reliable infection assay for high-throughput analysis of host response. Interestingly, the T3SS of *Y. pestis* has been found to be less efficient in cell culture compared to that of *Y. enterocolitica*[36,37]. A key mediator of *Yersinia* pathogenesis is the YopP/J effector, (YopP in *Y. enterocolitica* and YopJ in *Y. pestis*), which induces apoptosis in the host. Although YopP and YopJ share ~97% sequence identity, YopP exhibits a greater capacity for accumulation in the host cells, which correlates with enhanced cytotoxicity [23]. We speculate that the relatively weaker pathogenic effect of YopJ may have been the basis of difficulty in developing a robust RNAi screen using *Y. pestis*.

**Figure 8:**
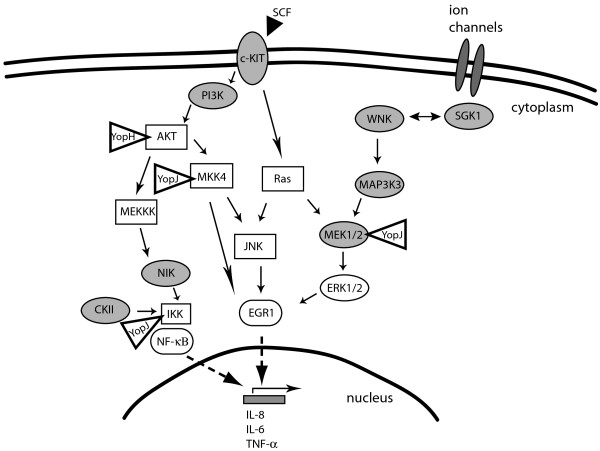
**Schematic of multiple signaling pathways induced by extracellular stimuli to activate transcription factors that regulate the pro-inflammatory cell response.** Cell surface receptors translate ligand binding into activation of host intracellular signaling pathways. The genes depicted in grey were identified in the RNAi screen in which gene silencing counteracted *Yersinia*-mediated inhibition of NF-κB activation in response to TNF-α. Cell stimuli, such as stem cell factor (SCF, black triangle), the natural ligand of c-KIT, initiate cell signaling that converge on the activation of two key transcription factors NF-κB and EGR1. Bolded triangles depict interactions between *Yersinia* Yop effectors and host signaling proteins. The cartoon includes primary signaling molecules and is not a comprehensive description of all known players or feedback mechanisms in these signaling pathways.

In this study, we describe a c-KIT-EGR1 signaling pathway that is targeted by *Yersinia* during infection. Although c-KIT and EGR1 have not been previously positioned experimentally in the same pathway to the best of our knowledge, c-KIT and EGR1 functions can be linked based on convergence of multiple overlapping pathways (Figure 8). Activation of c-KIT has been shown to stimulate the JNK, MEK/ERK, and PI3K/AKT signaling pathways, which can feed into EGR1 [30,31,38] and other transcription factors to regulate cell growth, differentiation and inflammatory responses [39,40]. In turn, EGR1 regulates expression of chemokines (e.g. IL-8, CCL2) and cytokines (IL-6, TNF-α) and was found to act synergistically with NF-κB to stimulate IL-8 transcription [41].

Our results support a model in which c-KIT signaling is targeted by *Yersinia* T3SS to suppress pro-inflammatory responses. Some kinases activated downstream of c-KIT, such as MEK and PI3K, have been shown to be inhibited by the *Yersinia* effectors YopJ and YopH, respectively [9,10,42]. YopJ has also been shown to inhibit phosphorylation of MKK4/SEK1 and attenuates JNK signaling and subsequent EGR1 activation [43] (Figure 8). Our findings suggest that downregulation of a receptor kinase function that leads to NF-κB activation can ameliorate the inhibitory effect of *Yersinia* T3SS. Since we observed that the inhibition of another signaling protein AKT1 also resulted in higher production of TNF-α by *Yersinia*-infected macrophage cells (Figure 3), we hypothesized that upon bacterial infection, multiple signal transduction pathways are triggered by various host extracellular and intracellular receptors of pathogen associated molecular patterns (PAMPs). However, not all signaling pathways are inactivated by *Yersinia* during infection, and inhibition of c-KIT may lead to redirection to alternative signaling pathways, such as the LPS-activated CD14 and TLR4 signaling to p38 and JNK, to recover NF-KB-driven gene expression [44,45]. This hypothesis is supported by our observations that pharmacological inactivation of JNK1 using the inhibitor BI-78D3 did not recover pro-inflammatory gene expression in THP-1 cells infected with pathogenic *Yersinia* (Figure 5A), while AKT1 and c-KIT inhibition resulted in increased TNF-α production in infected THP-1 and NHDC (Figure 3). Thus, redistribution of signaling pathways can still lead to mitigation of NF-κB-regulated immune response during the course of *Yersinia* infection.

The exact mechanism of *Yersinia* activation of c-KIT remains unclear. The natural ligand of c-KIT, SCF, has been shown to activate c-KIT phosphorylation within 5 min of treatment [34,35]. In response to *Y. enterocolitica*, c-KIT exhibited maximal phosphorylation at ~45 min post-infection in THP-1 cells by Western blot (Figure 6), demonstrating that *Yersinia* infection is capable of stimulating c-KIT activation, albeit via a delayed response compared to SCF. Since, we observed this delayed phosphorylation in both virulent and attenuated *Y. enterocolitica*, it may be the case that LPS or other bacterial cell surface molecule can mediate host receptor phosphorylation and/or signaling, rather than solely the T3SS. We have also shown that inhibition of c-KIT signaling by the small molecule OSI-930 induced an altered inflammatory gene expression pattern in response to pathogenic *Yersinia* that resembled infection by a non-virulent strain (Figure 5A), further supporting functional links between c-KIT activity and *Yersinia* virulence. It may be the case that Yop effectors either directly or indirectly modulate c-KIT function following injection into the host. In preliminary studies, we have found a strong binding interaction between c-KIT and the T3SS chaperone SycE (data not shown). Another possibility is that *Yersinia* interacts with lipid rafts containing c-KIT in the plasma membranes of host cells during the infection process [46,47]. Activation of receptor tyrosine kinases by bacterial LPS has been reported previously. For example, EGFR transactivation by LPS was induced by p38 and matrix metalloproteases upon TLR4-LPS interaction and was essential for COX-2 gene expression [48]. Increased phosphorylation of EGFR was observed 5–60 min of treatment with purified LPS.

In the search for host factors whose functions are required by pathogenic *Yersinia* to suppress the host innate immune response, we identified additional genes that belong to common functional networks. For example, the SGK and WNK families directly regulate each other to control osmotic stress and cellular ion balance. During *Yersinia* infection, the needle-like T3SS injects effector proteins into the host, increasing membrane permeability and introducing osmotic stress to the host [49]. Osmotic stress caused by ion imbalance can activate SGK1/WNK1 function and modulate downstream MAPK-ERK signaling pathways [50,51], thus potentially providing *Yersinia* with another signaling pathway to manipulate gene expression. WNK1 is a substrate of SGK1 during insulin activation of PI3K [52] and can activate SGK1 during ENaC regulation [53]. WNK1 also participates in an epidermal growth factor receptor (EGFR)-ERK pathway that includes two signaling molecules, MAP3K3 and MEK1/2, which were also identified as hits from our RNAi screen (Figure 8). A direct protein-protein interaction between WNK1 and MAP3K3 has been previously demonstrated [54]. MAP3K3 regulates ERK signaling through MEK1/2 and is required for NF-κB activation [55-57]. The *Yersinia* effector YopJ has been reported to catalyze the acetylation of target kinases to inhibit MEK and NF-κB signaling [9,10]. Similar to c-KIT inactivation, downregulation of WNK1 and MAP3K3 may shunt the activation of transcription factors that regulate inflammatory cytokine release to an alternative signaling pathway.

Several of the RNAi screen hits that impact signal transduction can be directly linked to regulation of NF-κB signaling. For example, the catalytic α subunit of CKII was found to phosphorylate IKKα with high specificity and to stabilize targeting of IκB for proteosomal degradation in response to such cell stressors as UV radiation and TNF-α [58-60]. NIK/MAP3K14 regulates the alternative NF-κB signaling pathway [61]. PIK3R2, a regulatory subunit of PI3K, functions in AKT activation, which leads to phosphorylation of p50 or activation of IKKα through multiple signaling pathways [61].

## Conclusions

Collectively, our studies have identified multiple host kinases, that when downregulated, mitigated *Yersinia*-mediated suppression of the host primary immune response. In particular, c-KIT is of great interest as a potential biomarker for susceptibility to *Yersinia* infection, given our preliminary data showing that primary dendritic cells that express higher c-KIT levels produced less TNF-α in response to *Y. pestis* infection. Furthermore, some of the identified genes and signaling pathways have been found to be essential for infection by other bacterial species. For example, the PI3K pathway is required for successful infection in *Yersinia* (this study), *Listeria* and *Salmonella*[13,62]. Thus, the RNAi screen hits may represent candidate targets for development of host-derived therapeutics that inhibit not only *Yersinia* infection, but also potentially a wide range of bacterial pathogens that employ common virulence mechanisms.

## Methods

### Tissue culture cell growth conditions and chemicals

The GloResponse™ NF-κB RE-luc2P-HEK293 cell line (Promega, Madison, Wisconsin), was cultured in DMEM (Invitrogen, Carlsbad, CA) supplemented with 10% FBS (HyClone, Logan, UT), 2 mM glutamine, 1 mM sodium pyruvate, and 50 μg ml^-1^ Hygromycin B (HygB) (DMEM/10-HygB). For the transfection assays, host cells were maintained in antibiotic-free DMEM/10% FBS. THP-1 human monocytes (ATCC TIB-202) were maintained in RPMI-1640/10% FBS. Normal human dendritic cells (NHDC) (LONZA, Allendale, NJ) were cultured in LGM-3 Growth Medium (LONZA). All media types do not contain any SCF, the natural ligand of c-KIT. All cell types were cultured at 37°C and 5% CO_2_. Phenol-purified lipopolysaccharide (LPS) from *E. coli* 055:B5 (Sigma-Aldrich, St. Louis, MO) was used as a positive control to induce cytokine release by host cells. The inhibitors TBB, H-89, CKI-7, and BI-78D3 were purchased from Sigma-Aldrich. OSI-930 was obtained from Selleck Chemicals (Houston, TX).

### Bacterial strains and growth conditions

The following *Yersinia* strains were used in this study: *Y. pestis* medievalis KIM5- (pCD1+, pgm-) [63], *Y. pestis* orientalis India195 (pCD1+, pgm+, LANL archive), *Y. enterocolitica* WA (pYV^+^, ATCC 27729), and *Y. enterocolitica* WA-01 (pYV^-^, this study). Strains were routinely propagated on brain heart infusion agar (Difco, Detroit, Mich) at 26°C overnight and up to 1 week storage at 4°C. For cell infection experiments, bacteria were grown at 26°C in brain heart infusion broth for 18 h in an orbital shaker at 180 rpm, followed by dilution of the bacterial culture to obtain 0.1 OD_660_ and additional growth for 2 h at 37°C (100 rpm). The pYV^-^*Y. enterocolitica* strain was obtained by serial passages of *Y. enterocolitica* WA on LB agar plates at 37°C. Bacterial clones were isolated and loss of pYV plasmid was monitored by PCR using primer sets for amplification of *yopH* and *yopJ*.

### RNAi screen and bacterial infection

The human GIPZ lentiviral shRNAmir kinome library, consisting of 2503 shRNA constructs targeting 782 genes, and 62 shRNA constructs isolated from the human druggable library selected to target 26 genes with heat shock or chaperone activity, were obtained from Open Biosystems (Thermo Scientific, Huntsville, AL). The shRNAmir libraries containing plasmid DNA were arrayed in 96-well plates such that each well contained one unique and identifiable shRNAmir. The library matrix was introduced into RE-luc2P-HEK293 cells using a high-throughput transfection method: 100–200 ng shRNA plasmid DNA was incubated at RT for 20 min in 20 μl serum-free MEM containing 600 nl TransIT-Express reagent (MirusBio, Pittsburgh, PA) and transfected into 2×10^4^ HEK293 cells in 100 μl DMEM/10% FBS. Approximately 30 h after transfection, culture media was replaced with DMEM/10% FBS containing 1 μg ml^-1^ puromycin. After 72 h of selection, during which >80% of the mock-transfected cells died, the selection media was removed, cells were washed with PBS, and then re-suspended in 200 μl serum-free DMEM containing 1 μg ml^-1^ trypsin.

The cell suspension (50 μl) was aliquoted to four white, clear bottom replica plates containing 50 μl DMEM/20% FBS. Cells were incubated 24h at 37°C prior to bacterial infection. For a more precise estimation of multiplicity of infection (MOI), one of the replica plates was used to calculate the number of host cells with the Cell Titer-Glo assay (Promega, Fitchburg, WI). A standard curve that correlates the ALUs to cell number (5000, 10000, 15000, 20000, 25000, and 30000 cells per well) was determined for every batch of substrate. Two of the three remaining replica plates were infected with *Y. enterocolitica* WA at MOI 5 by addition of bacteria in 5 μl DMEM/10% FBS, followed by centrifugation at 200 g for 5 min at RT. The remaining replica plate was used as a reference control (MOI 0). After 1h at 37°C, 20 μl DMEM/10% FBS containing 800 μg ml^-1^ of the bacteriostatic antibiotic chloramphenicol was added to each well in the plates to limit further *Y. enterocolitica* growth and to avoid activation of apoptotic pathways. Applying Cell Titer-Glo (Promega), we determined that the HEK293 cells infected with *Y. enterocolitica* at MOI 5 exhibited maximal inhibition of NF-κB-driven gene expression in response to TNF-α stimulation with no or minimal cellular toxicity.

At 5 h post-infection, 25 μl DMEM/10% FBS containing 50 nM TNF-α was added to all culture plates. The screen was run once in duplicate plates. At 20h post-infection, the Cell Titer-Glo assay was used to normalize NF-κB-driven luciferase activity to the cell titer. Arbitrary luciferase units (ALUs) were measured using the Synergy2 Multi-Mode Microplate Reader (BioTec, Winooski, VT). The relative percentage of NF-κB inhibition (R%I) by *Yersinia* infection was determined using the formula, R%I = [1-(ALU:MOI 5/ALU:MOI 0)]×100, where ALU:MOI 5 corresponds to the luciferase activity in bacteria-infected cells relative to ALU:MOI 0, the luciferase activity in no infection control.

### Hit selection criteria and validation assays

Genes with at least two shRNAmir constructs that resulted in ≥40% (≥ 2 SD) decrease in R%I of NF-κB reporter gene activity were chosen for further validation. Selected hits were analyzed using siGENOME SMART pool siRNAs from Dharmacon (Thermo Scientific). RE-luc2P-HEK293 cells (2.5 × 10^5^ per well) were transfected with a 10 nM siRNA pool of four sequences per target gene in a 96-well plate and cultured for 72 h prior to *Y. enterocolitica* WA and *Y. pestis* Ind195 infection at various MOI with or without TNF-α stimulation. Total RNA was isolated using the RNeasy kit (QIAGEN, Valencia, CA) following the manufacturer’s instructions. mRNA expression levels were determined by real-time quantitative PCR (qPCR) with TaqMan Gene Expression Assays and the TaqMan RNA-to-C_T_™ 1-Step Kit (Applied Biosystems, Foster City, CA) using a 7300 real-time cycler (Applied Biosystems). NF-κB-driven luciferase activity was quantified using the Cell Titer-Glo assay.

### ELISA and Luminex 200-based assays for analysis of cytokine levels

TNF-α cytokine levels were measured in the culture supernatant of *Yersinia*-infected THP-1 cells by ELISA (BD Biosciences, San Diego, CA) following the manufacturer’s instructions. Conditioned media was collected 24 h post-infection and passed through a 0.22 μm syringe filter for analysis. Cytokine levels in the supernatants of *Yersinia*-infected NHDC cultures were determined by Luminex Immunoassays using Human Cytokine 3-plex custom-made panels from Invitrogen (Life Technologies, Carlsbad, CA) and Procarta (Affymetrix, Santa Clara, CA) on the Luminex 200 platform (Luminex, Austin, TX).

### Gene expression assays

We utilized the RT Profiler Human Signal Transduction PathwayFinder PCR Array, PAHS-014A (SABiosciences/QIAGEN, Frederick, MD) to profile 84 genes that function in 18 different signal transduction pathways. Total RNA (1.5 μg) was isolated 24 h post infection using the RNeasy Miniprep Kit (QIAGEN) and 1 μg RNA transcribed into cDNA using the RT^2^ First Strand Kit (SABiosciences/QIAGEN) following the manufacturer’s recommendations. The cDNA reactions were added to RT^2^ SYBR Green ROX™ qPCR Mastermix (SABiosciences/QIAGEN) and redistributed on 96-well profiler array plates. Reaction mixtures were amplified and analyzed on a 7300 real-time cycler (Applied Biosystems). Dot plots represent array data normalized to beta-2-microglobulin and internal RT and PCR controls. Data analysis was performed using an Excel-based template provided by SABiosciences/QIAGEN. mRNA expression levels of, EGR1, VCAM1, CCL20, IL-8, NF-κB1, and RelA were determined by qPCR using TaqMan Gene Expression Assays (Applied Biosystems).

### Western blot analysis of c-KIT

THP-1 cells were infected with *Y. enterocolitica* at MOI 40 or stimulated with 50 ng/ml SCF (Cell Signaling Technology, Beverly, MA). Cells (3×10^6^) were harvested at the indicated time points, washed with PBS, and lysed in 1 ml buffer A (40 mM Hepes, pH 7.4, 1% Triton X-100, 1 mM EDTA, 150 mM NaCl, 50 mM NaF, 1 mM sodium orthovanadate, 10 mg/ml leupeptin, 10 mg/ml aprotinin, and 1 mM PMSF). Lysates were pre-cleared by incubation with 50 μl Protein A Sepharose (Invitrogen, Carlsbad, CA) for 1h at 4°C and centrifuged at 12,000 g for 15 min. c-KIT was enriched from whole cell lysates by overnight incubation at 4°C with 1 μg mAb against c-KIT (104D2, Santa Cruz Biotechnology, Santa Cruz, CA), followed by immunoprecipitation with 50 μl Protein A Sepharose for 1 hr at room temperature, and three washes in buffer A. Proteins were eluted by boiling in NuPAGE LDS Sample buffer (Invitrogen), separated by SDS-PAGE, and analyzed by Western blot using either c-KIT (104D2) or phosphorylated Tyr (PY20, Santa Cruz Biotechnology, CA) primary antibodies at 1:1,000 dilution. Blots were developed using rabbit anti-mouse antibody coupled to HRP at 1:10,000 dilution and the ECL detection system (Amersham/GE Healthcare, Piscataway, NJ). Densitometry of individual bands was quantified using the ChemiDoc XRS system (Bio-Rad, Hercules, CA). The 60 kDa fraction of IgG was used as an internal loading control, and the percentage of phosphorylated c-KIT was calculated based on the normalized data for both total and tyrosine phosphorylated c-KIT.

### RelA/p65 activation assays

THP-1 cells were incubated in media, with or without 1 μM OSI-930, for 5 h and then infected with *Y. enterocolitica* for 45 min at MOI 40. Cells were pelleted and incubated in hypotonic lysis buffer NB (10 mM Tris, pH 7.9, 1.5 mM MgCl_2_, 10 mM KCl, 0.5 mM DTT, 0.5% NP-40, 10 mg/ml leupeptin, 10 mg/ml aprotinin, and 1 mM PMSF) for 15 min on ice. Cell nuclei were purified by centrifugation on 30% sucrose in NB buffer at 800 g for 10 min and resuspended in PBS/3.7% formaldehyde. Fixed cell nuclei were blocked in PBS/10% goat serum/1% BSA/0.1% Triton for 1h, incubated with 1:300 dilution of mouse anti-phospho-NFκB p65 (A-8, Santa Cruz Biotechnology) for 3 h, followed by 1 h incubation in 1:500 dilution of goat anti-mouse IgG conjugated to FITC (Abcam, Cambridge, MA), all at room temperature. After five washes in blocking buffer, the nuclei population was analyzed on a FACS CaliburII (Becton Dickinson, Franklin Lakes, NJ) using a blue laser (488 nm) and 530/30 emission channel with CellQuest Pro software.

### Flow cytometry analysis of c-KIT levels on cell membranes

Formaldehyde (3.7%)-fixed NHDCs were rinsed with PBS containing 50 mM NH_4_Cl for 15 min. Cells were blocked with pre-immune heterologous serum (1:10 diluted in PBS) for 30 min, washed with PBS and incubated with primary phycoerythrin (PE)-conjugated c-KIT (Ab81, sc-13508PE, Santa Cruz Biotech, CA) for 4 h. The cell populations were acquired using a BD FACS CaliburII instrument with the blue laser (488 nm) and 585/42 emission channel and were analyzed using BD CellQuest Pro software.

### Statistical analysis

Paired two-tailed Student’s t-test was used to calculate p-values, where ≤0.05 was considered statistically significant. To evaluate the robustness of the RNAi screen in a high throughput setting, the Z-factor was calculated as Z’ = [1- (3(σ_p_ + σ_n_)/[ μ_p_- μ_n_])], where the mean (μ) and standard deviation (σ) of positive (p, infected with *Yersinia*), and negative (n, bacteria free) samples were applied. A standard z-score was used to identify hits from the RNAi screen. The z-score was based on a raw score defined as z = (x-μ)/σ, where x is a reporter gene activity from a single well, μ is the mean reporter gene activity calculated for entire plate including non-silencing shRNA samples, and σ is the standard deviation of the entire plate.

## Abbreviations

ELISA: Enzyme-linked immunosorbent assay; HTS: High-throughput screen; MAPK: Mitogen-activated protein kinase; MOI: Multiplicity of infection; NHDC: Normal human dendritic cells; qPCR: Quantitative polymerase chain reaction; RNAi: RNA interference; shRNA: Short hairpin RNA; siRNA: Small interfering RNA; T3SS: Type three secretion system; Yop: *Yersinia* outer protein.

## Competing interests

The authors declare that they have no competing interests.

## Authors’ contributions

SM-V designed and carried out the RNAi screen, the validation studies of the candidate genes, and drafted the manuscript. YS performed the *Y. pestis* studies. KN-L and KDR participated in the design of the study. EH-G conceived of the study, participated in its design and coordination, and helped draft the manuscript. All authors read and approved the final manuscript.
